# Editorial: Plant responses to environmental change

**DOI:** 10.3389/fpls.2026.1991928

**Published:** 2026-10-06

**Authors:** Shuangxi Zhou, Aoxue Wang, Yanchang Wang, Yuguo Liu, Cungang Cheng

**Affiliations:** 1 Department of Biological Sciences, Macquarie University, Sydney, Australia; 2 College of Horticulture and Landscape Architecture, Northeast Agricultural University, Harbin, China; 3 Wuhan Botanical Garden, Chinese Academy of Sciences, Wuhan, China; 4 Institute of Ecological Conservation and Restoration, Chinese Academy of Forestry, Beijing, China; 5 Institute of Pomology, Chinese Academy of Agricultural Sciences, Xingcheng, China

**Keywords:** abiotic stress, acclimation, adaptation, biotic stress, climate change, drought, heat stress, resilience

Plants can adjust to environment to optimize their fitness. Quantifying the genetical, morphological and/or functional modifications of plants, plant communities and ecosystems under environmental changes, and characterizing the underlying processes and mechanisms, are important for us to understand and assess their potentials in terms of stress resilience, adaptation, and acclimation. This Research Topic attracted 20 submissions from researchers worldwide in this area and published 10 of them.

By bridging genetic, molecular and physiological approaches, Yang et al. compared the gene expression patterns and trait-based resource acquisition strategies of ten native alpine tundra plant species and one encroaching species. The study highlighted that traditional stressors exerted lower regulatory pressure on the encroaching species, which showed efficient nitrogen use and cold insensitivity, explaining its successful encroachment into the tundra ecosystem. Yang et al. investigated the how low light modulated source-sink dynamics of saffron plants, affecting their carbohydrate synthesis and transportation and the corm yield. Li et al. highlighted the role of alternative splicing regulation in enhancing the stress adaptability of hot pepper. Zhao et al. reported insights on the complexity of grain quality and aroma volatiles formation of waxy maize under organic fertilizer substitution. Wang et al. constructed a transcriptome map of trifoliate orange under cold stress.

On the organ and whole-plant scale, Zhou et al. highlighted a decoupling relationship between multi-trait morphological modifications and foliar ion accumulation under prolonged salinity stress. Duan et al. investigated the impacts of seedling cultivation and transplanting methods on rice submergence tolerance. The study uncovered that submergence tolerance was affected by seedling cultivation and transplanting methods which regulated stress-indicating morphological and biochemical traits. On the provincial scale, Jiao et al. utilized a machine learning approach to map vegetation stability across Jiangsu Province. The study highlighted the key roles of radiation and vapor pressure deficit in regulating vegetation stability.

By bridging experiment with modelling, Ye et al. compared the accuracy of different photosynthetic models in representing observed plant photosynthetic responses under different oxygen concentrations. Qian et al. explored the environmental drivers and underlying molecular mechanisms governing bamboo shoot nutrition, and developed a predictive model linking environmental factors, gene expression, and nutritional traits.

The 10 articles published in this Research Topic provided up-to-date content and insights to the readers. However, we note that the observations in most articles were lab or glasshouse based. Further studies conducted in field or commercial farms, with multiple environmental factors, in the longer temporal scales, and on the interactions between abiotic and biotic stresses, would be worth investigating. Topics such as leveraging stress to improve crop performance would also be interesting.

